# *Citrobacter freundii* Foodborne Disease Outbreaks Related to Environmental Conditions in Yazd Province, Iran

**Published:** 2019-06

**Authors:** Farzaneh AMINHARATI, Mohammad Hassan EHRAMPOUSH, Mohammad Mehdi SOLTAN DALLAL, Mehdi YASERI, Abbas Ali DEHGHANI TAFTI, Zahra RAJABI

**Affiliations:** 1. Division of Microbiology, Department of Pathobiology, School of Public Health, Tehran University of Medical Sciences, Tehran, Iran; 2. International Campus, Shahid Sadoughi University of Medical Sciences, Yazd, Iran; 3. Department of Environmental Health Engineering, School of Health, Shahid Sadoughi University of Medical Sciences, Yazd, Iran; 4. Food Microbiology Research Center, Tehran University of Medical Sciences, Tehran, Iran; 5. Department of Epidemiology and Biostatistics, School of Public Health, Tehran University of Medical Sciences, Tehran, Iran; 6. Department of Disaster and Emergency Health, Shahid Sadoughi University of Medical Sciences, Yazd, Iran

**Keywords:** Climate, *Citrobacter freundii*, Environment, Foodborne disease, Outbreak, Iran

## Abstract

**Background:**

The aim of this study was to assess associations of *Citrobacter freundii* foodborne outbreaks with environmental factors in various regions of Yazd Province, Iran, 2012–2016.

**Methods:**

The public health surveillance data were used for one period of five years reported foodborne disease outbreaks in various regions of the Province. Multilevel regression statistical method was used to analyze associations of climatic and demographic variables with outbreaks. Significant associations were tested using likelihood ratio tests.

**Results:**

Results showed a significant association between *C. freundii* outbreaks and air dust conditions, age groups and various regional cities.

**Conclusion:**

The current study revealed necessity of etiologic agent identification for use in foodborne disease outbreak guidance in future outbreaks. Systemic surveillance schemes can help prevent and control similar scenarios using reports of environmental effects on foodborne disease outbreaks.

## Introduction

Foodborne outbreaks are defined when two or more unrelated people used the same contaminated food or drink and showed the same clinical symptoms (1). We investigated *Citrobacter freundii* foodborne disease outbreaks related to various climatic parameter and demographic factors in Yazd Province, Iran. *Citrobacter* spp*.* are facultative anaerobic, motile, gram-negative bacilli in the Enterobacteriaceae family that is widely distributed in environment and intestinal tracts of human and animals so these bacilli regarded as the environmental contaminants or harmless colonizers (2).

Severe gastroenteritis foodborne outbreaks attributed to *C. freundii* have been reported in 152 patient of Germany‘s institutional centers nursery school by using prepared food with green butter made of contaminated parsley from an organic garden (3). An enteric pathogen investigator in China’s Henan Province isolated the *C. freundii* strains from 12 diarrheal patients, 11 animals and insects that showed the strongest aggregative adhesion and cytotoxicity to *HEp2* cells (4). The last research on *C. freundii* outbreaks showed the most rates of foodborne disease during summer months and in adult patients, so we studied on the temporal climatic pattern and the regional demographic factor impacts on *C. freundii* food-borne disease outbreaks.

Climate variations ascribed directly or indirectly to human activities by the ranges of hazards and life conditions of the Earth changed during the prolonged period. The changes in environmental conditions can influence the health of human by the different situations according to space scales, so the vulnerabilities of population will be varies depending on the environmental topographies (5). The environmental impacts on ecological conditions of microorganisms caused new human disease outbreaks during the seasonal changes of surface water temperatures by the global climatic variations (6). Moreover, last research proved the effects of global desert dust transmissions in many environmental processes, so the pollutant weather and dust conveys have harmful roles on human’s health and ecosystems (7). Meteorological circulation of dust can change microbial densities for the long distance across the sea and mountain by varying environmental conditions (8).

Recently the new infectious disease reported that was related to dust hovering across the nations and continents in the world, similarly the transmission of *Citrobacter* spp. was previously detected as the air-particle associated (9). Although the environmental factors are the most reasons for original change in disease rates, the health facilities often have been playing an important role in prevention and control of disease outbreaks. Based on the fact that precipitation risk factor may cause runoff potential sources of contaminated watershed, human’s interface activities on microbial water qualities such as agricultural interventions can influence the water sanitary and food production safety (10, 11). Furthermore, the community resiliency and responses to intermittent climate extremes are not operative in gradual climatic events. Therefore, despite the intensifying food importation that may control foodborne disease during the drought in the world, the poor countries are not able to do such indeterminable proceedings in response to long-term drying of drought areas (12).

We assessed the health impacts of short-term climatic variation, which required the yearly continuous health data to estimate the burden of *C. freundii* foodborne disease outbreaks in Yazd Province, Iran.

## Materials and Methods

### Data Collection

This cross-sectional study was carried out in Yazd Province located in the center of Iran (longitude 54.4342 E and latitude 32.1006 N) with a population of more than 1,138,533 m and an area of 131,575 km^2^, 2012–2016. Surveillance team noted 729 databases of patients included households, place, and onset time of outbreaks, while the study on patients did not need the approval forms. The reported data was different from state-to-state and each outbreak contained essential information of demographics, medical history, symptoms, laboratory results, and treatment, hospitalization, food or water meals outdoor, indoor of the home and travel history of patients. The incubation period and clinical signs have been considered for each case of exposures (1). Demographic information was obtained from Iran’s statistical center and then classified according to age, genus, and type of community as the household and instituted social groups (13). As well as, the climatic data such as monthly mean of temperature, relative humidity, rainfall, and dust conditions was obtained from the national meteorological center ( http://www.irimo.ir/far/index.php ).

### Laboratory methods

The rectal swab of each patient was inoculated in Cary-Blair transport media (DIFCO, USA) and received by the Food Microbiology Research Center in Tehran University of Medical Sciences, Tehran, Iran. The Enriched samples was streaked on MacConkey agar and incubated at 37 °C overnight, and then suspected colonies of *Citrobacter* spp. were identified using biochemical tests such as KCN, lysine decarboxylase, TSI, urea, citrate, motile ornithine, indole reagent, carbohydrate fermentation (DIFCO, USA). The *C. freundii* ATCC 8090 was used for quality control and serodiagnostic test carried out using commercial kits (MAST, Merseyside, UK) (14–17).

### Statistical methods

Data were entered to EXCEL shite and imported to SPSS v.16.0 (Chicago, IL, USA) for statistical calculations. The statistical analyses were performed in Stata v.14.0 (Stata Corp, College Station TX, USA) (18). The seasonal climatic and demographic variables used as the independent variables and *C. freundii*, outbreak considered as a dependent variable. We used multilevel regression model to assess the relations between dependent and independent variables. The significant association was tested by a likelihood ratio test and *P*-value<0.05 definition (13).

## Results

The reference lab reported 57 cases of *C*. *freundii* foodborne disease from 22 *Citrobacter* outbreaks, 2012–2016. The number of outbreaks in females and males was almost equal per 100,000 of population. The results of analysis showed no significant relations between the type of communities, and genus with the incidence rate (IR) of *C. freundii* foodborne disease outbreaks. There was an important association between the IR of food-borne disease and age groups, while the children aged ≤ 5 have been at more significant risk of disease (Table 1).

**Table 1:** The IR of *C. freundii* foodborne disease based on the demographic variables

***Variable***	***Level***	***N***	IR^*^	95% CI^*^		***P value***
				***Lower***	***Upper***	
Genus	F	29	0.60	0.404	0.866	0.561
	M	28	0.69	0.457	0.994	
Age (yr)	≤ 5	7	3.06	1.23	6.3	0.002
	6–20	23	0.93	0.588	1.39	
	21–45	23	0.52	0.328	0.777	
	46–59	4	1.72	0.469	4.41	
	60	0	0	0	0	0
Type of community	Family_1_	38	0.26	0.181	0.35	0.183
	Social_2_C	19	0.60	0.36	0.933	

IR^*^: The incidence rate of disease, CI^*^: Confidence interval

1: Family, who used household food

2: Social communities, who used restaurant food or live in organizational community

**Fig. 1:**
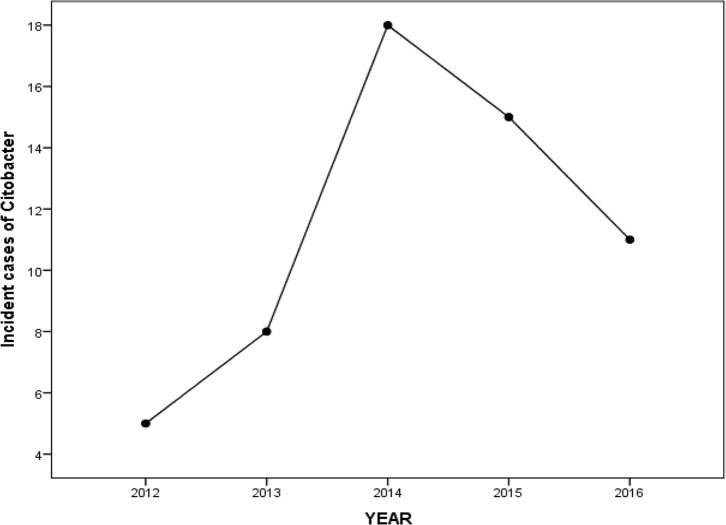
The incidence of *C. freundii* outbreaks based on the year

Moreover, we found that the IR of *C. freundii* foodborne disease outbreak was more in 2014 (Fig. 1). Furthermore, the significant relationship was between suspended dust condition and IR of *C. freundii* outbreaks, while the IR of outbreaks was 13.50 times by the external dust and 12.13 times by the inner dust. The IR of outbreaks reached to 1.17 times by each millimeter of rainfall. There was no association between temperature, humidity, and seasons with IR of outbreaks (Table 2). The analysis results revealed more obvious associations between the IR of outbreaks and different cities. The IR of outbreaks was in Ashkezar 4.43 times, Taft 6.63 times, and Ardekan 3.73 times compared to Yazd Reference City (Table 3).

**Table 2:** Incidence rate ratio of outbreaks based on the seasons and climatic parameters

***Variable***	IRR^*^	95%CI^*^		***P value***
		***Lower***	***Upper***	
Temperature	0.69	0.84	1.9	0.56
Humidity	0.92	0.83	1.00	0.74
Rainfall	1.17	1.067	1.28	0.001
Normal	Ref			
6	13.50	3.35	54.26	<0.001
7	12.13	3.11	47.28	<0.001
Seasons	Ref			
Summer	3	0.81	11.8	0.099
Fall	2.33	0.60	9.02	0.22
Winter	1	0.20	4.95	1.00

IRR^*^: Incidence rate ratio, CI^*^: Confidence interval, 6: Dust conditions originated from outside of the cities, 7: Dust conditions originated from inside of cities

**Table 3:** The Incidence rate ratio of outbreaks based on different cities in Yazd Province, Iran

***City***	IRR^*^	***P value***	95% CI^*^	
			***Lower***	***Upper***
1	Ref			
2	4.43	0.049	1.0076	19.507
3	1.22	0.848	0.1603	9.270
4	6.63	0.003	1.904	23.062
5	0	0	>1000	0.997
6	0	0	>1000	0.997
7	1.89	0.536	0.249	14.423
8	3.73	0.007	1.434	9.719
9	1.02	0.981	0.135	7.793

IRR^*^: Incidence rate ratio, CI^*^: Confidence interval, 1: Yazd Ref. City, 2: Ashkezar, 3: Mehriz, 4: Taft, 5: Mybod, 6: Abarkuh, 7: Khatam, 8: Ardekan, 9: Behabad

## Discussion

This study was the first research about the environmental impacts of climatic parameters on foodborne disease outbreaks in Iran that used multilevel regression method to quantify the relationships of climatic and demographic variables with *C. freundii* foodborne disease outbreaks. Although the *Citrobacter* spp. reported as a normal intestinal microflora, the investigations in Iran showed some antibiotic resistance isolated species from hen’s eggs and day-old chicks that induced diarrheal diseases (19). Furthermore, *Citrobacter* spp. found as fecal pollution of small community drinking water sources such as hospitals in different seasons of Tehran, Iran (20). Iranian last studies in 2014 revealed the increasing rate of Shiga toxin producing *Citrobacter* spp. o4.84% that caused intestinal disorders in three separate foodborne disease outbreaks (21). The investigations on 1–36 m children in university hospital of Naples reported 46 isolated *Citrobacter* spp*.* of 238 stool specimens, 1983–1984 (22). We isolated 57 *C. freundii* foodborne diseases of 729 rectal swaps, 2102–2016, while the children aged ≤ 5 years have been more significant vulnerable than other groups. The result of our study showed no significant relations between the seasons, months, and temperature with *C. freundii* foodborne disease outbreak IRs by statistical analysis model, while the most outbreaks happened in summer (3). By delving into analysis outputs, we found that atmospheric dust conditions have been in significant relationships with *C. freundii* outbreaks and the IR of outbreaks enhanced during summer in regional cities of province such as Ashkezar. Therefore, the increasing rates of outbreaks might be related to etiological agent of disease in air suspended dust, since the *Citrobacter* strains reported as the clinically relevant bacteria with warning worth occurrences in dust particles (9).

The more important reasons for increasing rate of *Citrobacter* foodborne disease outbreaks in Taft and Ardekan have been related to poor-hygienic quality of water that might be used for farm irrigation, or influencing the contaminated regional ground water or surface water by the human wastewater. As regards, the last investigations on gastrointestinal diseases in Mexico City and Pakistan reported the poor quality drinking water that caused health problems by the *Coliform* infections (23–25). *C. freundii* can be distributed in environment and its strains are linked to sporadic outbreaks of diarrheal disease in the world, que the *C. freundii* strains have been isolated from neonatal care unit of a large hospital. By this means, the *C. freundii* like as the *Enterobacter asburiae* and *E. cowanii* can be cause of common (NICU) infections in neonatal and high-risk groups in Iran (26–28). Furthermore, many of the new categorized Gram-negative bacteria in Enterobacteriaceae family that found in infant formula milk caused foodborne disease in Iranian infants, so the increased rate of foodborne disease in children ≤ 5 ages might be related to food poor safety (29).

## Conclusion

Environmental parameters such as air dust pollution can significantly increase the IR of *C. freundii* foodborne disease outbreak in more vulnerable aged groups. Therefore the epidemiological studies should be carried out to subtyping the resistance strains of *C. freundii* particularly in children aged ≤ 5 years. Moreover the poor quality of food safety should be considered in enhancing rate of foodborne disease outbreaks, so the strong institutional framework is needed to monitor the food safety.

## Ethical considerations

Ethical issues (Including plagiarism, informed consent, misconduct, data fabrication and/or falsification, double publication and/or submission, redundancy, etc.) have been completely observed by the authors.
